# Annotations

**Published:** 1908-09-26

**Authors:** 


### Annotations.

### Charitable Swindles.

The careless almsgiving which does so much credit to the heart and so little to the brain of the British nation has been exemplified anew in the frauds connected with the so-called "Pastor Housley's Orphanage," which have been investigated by the Blackpool magistrates. The "orphanage" was at first non-existent and never was an institution worthy of the name. The officials were a set of dishonest and disreputable swindlers. Yet apparently there was not much difficulty found in luring money out of the pocket of the public for a place of which they knew nothing. Collectors were paid a handsome commission on their takings, and doubtless were energetic in pushing the claims of the orphanage, but the money they handed in was in many cases unaccounted for. It was only after a dismissed official communicated with the police that the swindle was exposed. None of the contributors seem to have taken the trouble to visit or inquire about the place. The former is, perhaps, impossible for many people who give to what they believe to be charities; but surely the latter is not impossible. Clergymen or other men of repute can be applied to for an opinion of the merits of an institution which asks the support of the charitable public. If the place has existed for some time a financial statement should be possible. If no guarantee of honesty is forthcoming it would be better to refuse to give. Perhaps a deserving but struggling institution here and there may suffer, but, on the whole, the result is more likely to be that some clever swindlers who now fleece the charitable for their own interests would be compelled to try other ways of making a living. There are plenty of *bonâ fide* charitable institutions whose antecedents are known, so that the tender-hearted need not shrink from refusing to give to those of which they know nothing. Let them send the donation which they contemplated giving to the latter to one of the former. There is so much need for help for real charities that it is a pity to throw away good money on fraudulent imitations.

### The Cholera Epidemic.

At the time of writing no case of cholera has been reported within these islands, notwithstanding the alarming outbreak of that scourge in St. Petersburg; and it is to be hoped that we shall escape a visitation of the epidemic. There is, indeed, far less cause for alarm on this head now than there might have been only a few years ago, for the advances of sanitary science, and of the estimation in which it is held by the community, during the past two or three decades have been really very great. It has to be remembered that no epidemic of this essentially water-borne disease has prevailed in Great Britain for over forty years; and even should sporadic cases be imported hither from abroad, the vigilance of port sanitary authorities and medical officers of health, together with improved water supplies and general hygiene, are almost a complete guarantee against any serious outbreak. Still, there are districts and towns where a good deal is to be desired from a sanitary standpoint; and if the news from Russia induces a few of the defaulting local authorities to pay attention to the counsels of their medical experts and to set their houses in order, at least someone will profit by our neighbour's calamities. The avenues for the dissemination in this country of cholera from St. Petersburg are chiefly our east-coast ports, and anyone who has witnessed the commercial harbour of the capital of Russia at this time of year can appreciate in some degree how careful the watch must be. The Neva is frozen for some five months in the year and unnavigable, owing to floating ice, for some time longer, so that into the remainder of the year is concentrated the whole of the very extensive timber trade in which large numbers of British ships are engaged. The situation therefore is one which, faced with calmness and determination, there is no reason to be afraid of. Our defences we believe to be sufficient to protect the country from invasion, but there is no harm in redoubling precautions which in normal times are sometimes reduced to mere formalities. The medical profession, while reassuring the public on the prospect, must at the same time spare no pains to render that assurance trustworthy. The rigid enforcement of the health laws at our ports, the promptest isolation of any case in the least suspicious, and the greatest possible vigilance in diagnosis are duties which we owe to the State, and in which we believe we shall not be found lacking.

### Indecent Advertisements.

The report of the Select Committee, which was appointed to join with a Committee of the House of Commons to inquire into the law relating to indecent advertisements and pictures, has at length been issued. It is satisfactory to find that they have not disregarded the weak spot, of which offenders in this respect invariably avail themselves. In the first place, the publication of an obscene book is not an offence which can be dealt with summarily, although it is an indictable misdemeanour. In the second place, the law does not hold that advertisements of medicines for procuring abortion, or of appliances for preventing conception, are indecent. The Committee recommends that both these shortcomings be remedied. Such advertisements should be held illegal, and the offenders should be dealt with summarily. Perhaps the best feature of the Report is the broad-minded view taken by the Commissioners. They suggest that in all such cases far more should be left to the discretion of the magistrate in dealing with the matter. They hope, doubtless, that ordinary public opinion should find expression more through the good sense of the magistrate than through a fixed code, which may possibly allow escape by means of verbal quibbles. It is certainly time that the open advertisement of obscenity should be impartially eradicated. There is no need for a fanatical campaign, but there is room for a proper assertion of that sense of "good form" which is perhaps one of the elements in our greatness as a nation.

